# Optimal Feature Selection-Based Dental Caries Prediction Model Using Machine Learning for Decision Support System

**DOI:** 10.3390/bioengineering10020245

**Published:** 2023-02-13

**Authors:** In-Ae Kang, Soualihou Ngnamsie Njimbouom, Jeong-Dong Kim

**Affiliations:** 1 Department of Computer and Electronics Convergence Engineering, Sun Moon University, Asan-si 31460, Republic of Korea; 2 Department of Computer Science and Engineering, Sun Moon University, Asan-si 31460, Republic of Korea; 3 Genome-Based BioIT Convergence Institute, Sun Moon University, Asan-si 31460, Republic of Korea

**Keywords:** disease dental caries, gradient boosting decision tree, feature selection, machine learning, feature importance

## Abstract

The high frequency of dental caries is a major public health concern worldwide. The condition is common, particularly in developing countries. Because there are no evident early-stage signs, dental caries frequently goes untreated. Meanwhile, early detection and timely clinical intervention are required to slow disease development. Machine learning (ML) models can benefit clinicians in the early detection of dental cavities through efficient and cost-effective computer-aided diagnoses. This study proposed a more effective method for diagnosing dental caries by integrating the GINI and mRMR algorithms with the GBDT classifier. Because just a few clinical test features are required for the diagnosis, this strategy could save time and money when screening for dental caries. The proposed method was compared to recently proposed dental procedures. Among these classifiers, the suggested GBDT trained with a reduced feature set achieved the best classification performance, with accuracy, F1-score, precision, and recall values of 95%, 93%, 99%, and 88%, respectively. Furthermore, the experimental results suggest that feature selection improved the performance of the various classifiers. The suggested method yielded a good predictive model for dental caries diagnosis, which might be used in more imbalanced medical datasets to identify disease more effectively.

## 1. Introduction

Oral health is essential for general health and quality of life. Oral health means being free from throat cancer, infections and sores in the mouth, gum disease, tooth loss, dental caries, and other diseases so that no disturbances limit biting, chewing, smiling, speaking, and psychosocial well-being. One of the most occurring oral health diseases is dental caries. Dental caries is a health issue caused by residual food that attaches to the teeth, causing calcification. Teeth become porous, hollow, and sometimes fractured as a result. Dental caries is a disease associated with the hard tissues of the teeth, namely, enamel, dentin, and cementum, in the form of decayed areas on the teeth, occurring as a result of the process of gradually dissolving the mineral surface of the teeth and continuing to grow to the inside of the teeth.

According to Global Burden studies in 2019, dental caries is the most frequent oral illness impacting around 3.5 billion individuals, with 2 billion suffering from permanent dental caries [1]. Moreover, in 2020, more than 6 million patients with dental caries in the Republic of Korea visited dentists, of which 1.45 million were children (0~9 years old) [2]. Therefore, dental caries illness is a challenge for scientists to address.

Recent research on dental caries was conducted by Rimi et al. [3], who discussed the prediction of dental caries using machine learning (ML). In their experiments, they used nine algorithms, namely, K-Nearest Neighbor (KNN), logistic regression (LR), support vector machine (SVM), random forest (RF), naïve Bayes (NB), classification and regression trees, multilayer perception (MLP), linear discriminant analysis (LDA), and adaptive boosting (AdaBoost). The best results were obtained through LR, with an accuracy of 95.89%.

Meanwhile, to detect dental caries, researchers use data images, such as those produced by Zhang, X. et al. [4], Lee, S. et al. [5], Estai, M. et al. [6], Lee, J.-H. et al. [7], and Megan Leo et al. [8]. Zhang, X. et al. [4] used the convolutional neural network model method to classify 3932 images of dental caries. They managed to obtain a value of 0.8565 for the area under the curve (AUC). In their research, Lee, S. et al. [5] proposed a U-shaped Deep Convolution Neural Network (U-Net). The highest sensitivity value was 0.9372. Estai et al. [6] proposed the Inception-ResNet-v2 model to classify as many as 2468 images. The image dataset sample size was less than that in the study by. Lee, J.-H. et al. [7], which classified 3000 images using the transfer learning method based on the InceptionV3 model. Similar to [7], Megan Leo et al. [8] employed a Google Net inception V3 Convolutional based deep learning method to detect the presence of cavities in images. The noise in the input images was reduced using the selective median filter method and achieved an accuracy of 86.7%.

The study by Karhade et al. [9] predicted the prevalence of Early Children Caries (ECC) in infants through clinical, demographic, behavioral, and parent-reported oral health status. A set of 10 ECC predictors closely related to ECC induction were deployed with AutoML on Google Cloud. Via the survey recorded data, experimentation was conducted to detect dental caries, and ECC classification accuracy (area under the ROC curve (AUROC), sensitivity, and positive predictive value) was evaluated. As a result of the study, the model performance of single-item reporting was the highest, with an AUROC of 0.74, a sensitivity of 0.67, and a positive predictive value of 0.64. In a similar case study, Ramos-Gomez et al. [10] identified variables for induced dental caries in infants 2–7 years of age living in Los Angeles. An RF algorithm was trained to identify dental caries predictors. The most influential variables were the parent’s age (MDG = 2.97, MDA = 4.74) and the presence or absence of dental health problems in infants within 12 months (MDG = 2.20, MDA = 4.04).

You-Hyun Park et al. [11] proposed a predictive model for caries in infancy utilizing the LR, XGBoost (version 1.3.1), RF, and LightGBM (version 3.1.1) algorithms. Feature selection was performed through regression-based reverse removal and an RF-based permutation importance classifier. The results of this study showed that LR had an AUROC value of 0.784, XGBoost had an AUROC value of 0.785, RF had an AUROC value of 0.780, and LightGBM had an AUROC value of 0.78.

The efficiency of data modeling varies greatly depending on the type of processing and the ML model used. The preprocessing technique employed to identify the essential characteristics of a particular problem is referred to as feature selection, frequently performed for two reasons: to reduce dataset dimensionality and to tailor the dataset to suit the selected analysis method best. Input dimensionality reduction can increase performance by lowering learning time, reducing model complexity, and enhancing generalization capacity. The selection of appropriate characteristics can help reduce measurement costs and increase problem comprehension [12].

S. Memiş et al. [13] investigated and proposed a new kNN algorithm, Fuzzy Parameterized Fuzzy Soft kNN (FPFS-kNN), which is based on several pseudo-metrics of fuzzy parameterized fuzzy soft matrices (fpfs-matrices). FPFS-kNN can take into account the effects of parameters on classification by employing the pseudo-metrics of fpfs-matrices—a novel approach. FPFS-kNN also finds the nearest neighbors for each pseudo-metric and classifies data using the previously mentioned multiple distance functions. They conducted an experimental study with 35 UCI datasets to demonstrate the suggested method’s effectiveness in classifying data, and they compared it to the state-of-the-art kNN-based and non-kNN-based algorithms. Through five-fold cross-validation, all algorithms were trained and tested for ten runs. The findings of FPFS-kNN were then compared to those of the others in terms of the most often used measures, such as accuracy, precision, recall, micro F-score, and macro F-score. Following that, they presented experimental and statistical results showing that the proposed FPFS-kNN, designated as FPFS-kNN (P) and based on Pearson’s correlation coefficient, outperforms the state-of-the-art kNN-based algorithms in 24 of 35 datasets in terms of each studied measure and 31 of 35 datasets in terms of the accuracy measure. Furthermore, the results demonstrate that FPFS-kNN (P) outperforms the others in 29 datasets for accuracy and macro F-score rates and in 24 datasets for precision, recall, and macro F-score rates.

Over the last few years, gene expression data have been widely used with ML and computational tools. Several matrix-factorization-based dimensionality reduction algorithms have been developed in gene expression analyses. However, such systems can be improved in terms of efficiency and dependability. The research conducted by Farid Saberi-Movahed et al. [14] proposed a Dual Regularized Unsupervised Feature Selection Based on Matrix Factorization and Minimum Redundancy (DR-FS-MFMR), a new approach to feature selection. The primary goal of DR-FS-MFMR is to remove unnecessary features from the original feature set. To achieve this goal, the primary feature selection problem is stated in terms of two aspects: (1) the data matrix factorization in the feature weight matrix and the representation matrix and (2) the correlation information connected to the selected feature set. The objective function is then enhanced by applying two data representation features and an inner product regularization criterion to complete the redundancy minimization process and the sparsity task more precisely. A vast number of experimental investigations on nine gene expression datasets were carried out to demonstrate the competency of the DR-FS-MFMR approach. The computational results show that DR-FS-MFMR is efficient and productive for gene selection tasks.

The research conducted by Saeid Azadifar. et al. [15] proposed a unique graph-based relevancy–redundancy gene selection method for cancer diagnosis approaches that can effectively eliminate redundant and irrelevant genes. The proposed method uses a unique algorithm that finds gene clusters (i.e., maximum-weighted cliques). By grouping similar genes, the proposed method prevents the selection of redundant and similar genes. The provided findings show that the performance of the created similarity measure outperforms all other measures. On a colon dataset, the classification accuracy was 87.89%; on a Small-Round-Blue-Cell Tumor (SRBCT) dataset, it was 83.19%; on a Leukemia dataset, it was 92.14%; and on a Prostate Tumor dataset, it was 83.73%.

The main contributions of this study are as follows: to identify the most important characteristics needed to improve the detection of dental caries and to obtain the most performant model for dental caries prediction via comparative experimentation on a novel combination of *GINI* and *mRMR* techniques, which allow imbalanced dental data with a high number of features to be classified under GBDT selection to achieve efficient performance. The ML model described in this study efficiently provides patients with superior-quality dental services, including diagnosis and treatment.

The rest of this paper is structured as follows: Section 2 describes the caries prediction model proposed in this study. The experimentation and results are presented in Section 3. Section 4 presents the discussion, while Section 5 concludes the paper.

## 2. Materials and Methods

**Figure 1:**
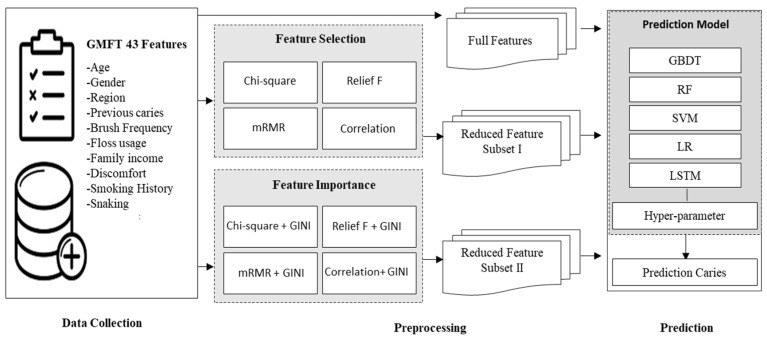
Proposed model for dental caries prediction.

This section presents the dataset, data preprocessing, an overview of the different methods used in this study, and the proposed prediction model employed for predicting dental caries. Five prediction methods were trained using the reduced subsets of features obtained via feature selection algorithms, and the performances of the ML methods were compared. A conceptual representation of the proposed dental caries prediction model is shown in Figure 1. Following data collection via the GMFT survey, a preprocessing operation was performed in which feature selection (*Chi-Square*, *Relief F*, *mRMR*, and *Correlation*) and feature importance (*Chi-Square + GINI*, *Relief F + GINI*, *mRMR + GINI*, *Correlation + GINI*) methods were used to curate the dataset and obtain the optimal dataset. Finally, the prediction algorithms were fine-tuned, and experimentation was carried out. The best performer was chosen as the final classifier.

### 2.1. Data Collection

The dataset used in this study is the 2018 Children’s Oral Health Survey conducted by the Korea Centers for Disease Control and Prevention. The data were collected by dentists visiting each institution for an oral examination survey. A total of 22,288 respondents were surveyed, and the oral health awareness questionnaire was selected and used for the survey. The oral health questionnaire consisted of a total of 43 items and 1 label, including age, gender, place of residence, snack frequency, tooth brushing frequency, oral care use, smoking experience, oral health awareness, and behavior, with act_caries as the label. These data are not subject to Institutional Review Board (IRB) approval, as they do not record patients’ personal information. Complete descriptions of the questionnaire items are available in Table S1 (see the Supplementary File).

### 2.2. Data Preprocessing

Data cleaning before building ML models is an essential step in increasing the efficiency and accuracy of the models [16], hence preventing bias or degradation of the model performance. The raw data were stripped of any empty or less critical features. The most significant features were chosen using feature importance and feature selection techniques (the methods used are described in the Supplementary File). Features that were redundant, less significant, or unrelated to the target variable were discarded, leaving just the optimal features. The selected optimal features were scaled (using the *Min–Max* algorithm) to values between 0 and 1 to improve training speed and model performance and to facilitate more effective learning and the understanding of the task.

**Definition 1.**  *The normalization of u is defined by*

(1) $$\hat{u_{i}}:=\frac{u_{i}-\underset{i}{\min}u_{i}}{\underset{i}{\max}u_{i}-\underset{i}{min}u_{i}}$$

**Figure 2:**
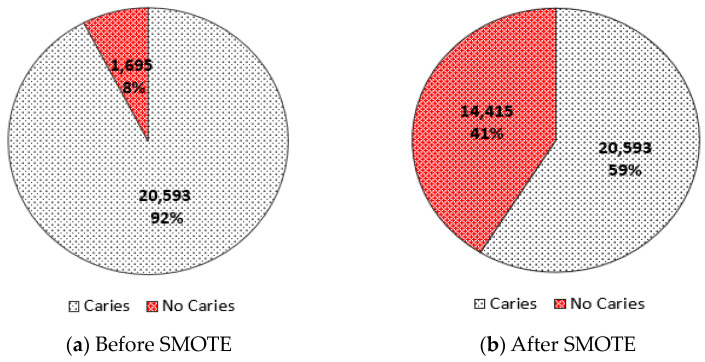
Data distribution ratio with respect to the target variable: (**a**) before SMOTE; (**b**) after SMOTE.

In Equation (1), $\hat{u_{i}}$ is the transformed value, $u_{i}$ is the original value, $\underset{i}{\min}u_{i}$ is the minimum value of the column, and $\underset{i}{\max}u_{i}$ is the maximum value. The *Min–Max* method reacts quickly in the presence of outliers and does not change the original content of the data. This study adopted the Synthetic Minority Oversampling Technique (*SMOTE*) algorithm, considered the most widely used oversampling technique employed to generate synthetic data [17]. This technique solves the problem of class imbalance present in the experimental data, therefore increasing the classification accuracy by solving the biased problem [18]. Our dataset contained an uneven data distribution, with almost 20,593 samples representing patients without dental caries “labeled 0” and 1695 samples for those with dental caries “labeled 1,” as seen in Figure 2a. The data distribution ratio with reference to the target variable before and after the applied SMOTE is shown in Figure 2.

### 2.3. Methods Used in the Proposed Prediction Model

This section describes the mRMR feature selection techniques, the GINI feature importance, and the GBDT algorithms. These were the three methods utilized in the design of the proposed model.

#### 2.3.1. Minimum Redundancy–Maximum Relevance (mRMR)

The features are rated according to their relevance to the target variable in the *mRMR* feature selection approach. The redundancy of the features is taken into account when ranking them. The feature with the highest rank in *mRMR* is the one with the most relevance to the target variable and the least redundancy among the characteristics. Redundancy and relevance are measured using *Mutual Information* (MI) [19,20,21].

**Definition 2.**  *Joint entropy is defined by*

(2) $$\;I\;\left(X,\;Y\right)=\sum_{y\in Y}\;,\;\;\sum_{x\in X}\left(x,y\right)log\left(\;\frac{p\left(x,y\right)}{p_{1}\left(x\right)p_{2}\left(y\right)}\;\right)\;$$

In this equation, p(x,y) signifies the compound probability distribution function of X and Y random variables, whereas $p_{1}\left(x\right)$ and $p_{2}\left(y\right)$ define the marginal probability distribution function of X and Y random variables, respectively. When two random variables are totally independent, the mutual information is *0*. It is symmetrical and cannot have a negative value (I(X,Y) ≥ 0, I(X,Y)=I(X,Y)). Assume that S is the feature to be chosen and that |S| denotes the number of items in this collection. The two criteria listed above must be met to ensure that the feature set chosen is the most efficient set [12].

**Definition 3.**  *The mutual information between the targeted class and feature is defined by*

(3) $$\;MI_{f\cdot t}(S,\;t)=\frac{1}{\left|S\right|}\;\sum_{F_{i}\in S}I\left(F_{i},t\right)$$

**Definition 4.**  *The mutual information between a defined feature is defined by*

(4) $$MI_{f\cdot f}=\frac{1}{{\left|S\right|}^{2}}\;\underset{F_{i}F_{j}\in S}{\sum }I\left(F_{i},\;F_{j}\right)$$

#### 2.3.2. GINI

*GINI* impureness (or the *GINI* index) is a statistic utilized in decision trees to determine how to divide the data into smaller groups. It estimates the frequency with which a randomly selected element from a set would be erroneously classified if randomly labeled according to the distribution of labels in a subset [22]. *GINI* importance (also known as the mean decrease in impurity) is one of the most often used processes for estimating feature importance. It is simple to implement and readily available in most tree-based algorithms, such as RF and gradient boosting. The *GINI* significance of a feature gauges its effectiveness in minimizing uncertainty or variance in decision tree creation. Thus, each time a split occurs in a tree regarding a particular characteristic, the *GINI* impurity is added to its total importance [23].

#### 2.3.3. Gradient Boosting Decision Tree (GBDT)

Gradient boosting is a type of ML technique used for regression and classification problems, with its weak prediction model (typically the decision tree) generating a forecast model in the form of a collection. It, like other strengthening approaches, constructs the model in stages and allows for the optimization of the loss function of any separable variables to a generalized model [24,25,26].

**Definition 5.**  *The initial constant value C  is obtained as*

(5) $$f_{0}=\underset{c}{\;argmin}\sum_{i=1}^{N}L\left(y_{i},\;C\right)$$

*where L(*$y_{i\;}$*C) is the loss function.*$arg_{c}^{min}$.

**Definition 6.**  *For each j = 1, 2, …, J, the residual along the gradient direction is written as*

(6) $$r_{im}=-{\left|\frac{\partial L\left(y_{i},\;f\left(x_{i}\right)\right)}{\partial f\left(x_{i}\right)}\right|}_{f\left(x\right)=f_{m-1}\left(x\right)}$$

**Definition 7.**  *The corresponding optimal fitting value for each j = 1, 2, …, J, is computed as follows:*

(7) $$\gamma_{jm}=\underset{Y}{\underset{︸}{\arg min}}\underset{x_{i}\in R_{jm}}{\sum }L\left(y_{i},\;f_{m-1}\left(x\right)+\gamma \right)$$

**Definition 8.**  *The following depicts the model update operation*:

(8) $$f_{m}\left(x\right)=f_{m-1}\left(x\right)+\overset{J}{\underset{j=1}{\sum }}\gamma_{jm},\;I\left(x\in R_{jm}\right)$$

**Definition 9.**  *The final model is obtained by*

(9) $$\;f\left(x\right)=f_{m}\left(x\right)=f_{0}\left(x\right)+\sum_{m=1}^{M}\overset{j}{\underset{j=1}{\sum }}\gamma_{jm}\;I\left(x\in R_{jm}\right)$$

### 2.4. Dental Caries Prediction Model

This section describes the proposed prediction model. The model receives three parameters: the training dataset *S*, the number of features to be considered, and the threshold of feature importance to be considered. At every iteration of the set of features, the weight importance of the feature is computed, and the *mRMR* is used to select the subset of features from the feature in *S*. The *mRMR* is calculated to rate the features according to their relevance to the target feature while considering redundancy. The features are ranked according to their *mRMR*, and a subset of features is selected according to the specified threshold. From the selected subset of features *H*, a sub-subset of features is selected via *Gini Impurity* (*GI*).

Four machine learning algorithms (RF, LR, SVM, and GBDT) and one Deep Learning (DL) model, Long Short-Term Memory (LSTM), are experimented on using the sub-subset features. RandomizedSearchAlgorithm is employed to find the best hyperparameters and reduce unnecessary computation, therein solving the drawback of GridSearchCV. Once the best parameters are found, training is performed. The best classifier (GBDT) is obtained after comparisons of the model evaluation metrics (accuracy, F1-score, recall, precision, and receiver operating curve (AUROC)). The complete algorithm of the proposed model is presented in Algorithm 1.
**Algorithm 1:** Proposed model for Dental Caries Prediction.**Input:** Training dataset S := ($x_{1}$,$\;y_{1}$), ($x_{2}$, $\;y_{2}$), …, ($x_{n}$,$\;y_{n}$); $F_{n}$ Number of features to be selected, and θ threshold of feature importance. **Output: highest performing classification approach C.** **Begin algorithm:**$for\;(feature\;f_{i}$ in S, i+1): Compute the weight importance of features. *relevance* = mutualInformation ($f_{i}$, y) *redundancy* = initialize to *0* $for\;(feature\;f_{j}$ in S, j+1) redundancy += mutualInformation (fi, fj) ***end for*** $mrmrValues\left[f_{i}\right]=$
*relevance*
−redundancy; 3.***end for***4.H=sort(mrmrValues).take(θ) select a subset of the most important features from the total set S.5.$for\;(feature\;f_{k}$ in H, i+1): rank features by their predictive power and select the most important one.Compute the Gini impurity of the target variable on $f_{k}$ as GI = $GiniImpurity\left(f_{k}\right)$.Split the data samples x from $f_{k}$ into two subsets $x_{1\sim k}$ and $x_{k\sim n}$ then compute the Avg Gini impurity of the two subsets. $${\bar{G}}_{avg\_1}=AVGGiniImpur(x_{1\sim k})$$$${\bar{G}}_{avg\_2}=AVGGiniImpur(x_{k\sim n})$$Store in the list of feature importance the Gini impurity caused by the splitting of the data on the current feature.6.Normalize the feature importances so that they sum to 1.7.Select a final subset of features based on feature importances obtained.8.Features = Select($\mathrm{List}{\_}_{feature\_importance}$, $F_{n}$)9.**PERFORM** the Caries Prediction
  Hyper-parameter  Machine Learning model include:   $\mathrm{List}{\_}_{Classifiers}$: GBDT, RF, SVM, LR, LSTM  ***for***
$C_{i}$ in $\mathrm{List}{\_}_{Classifiers}$:     $C_{i}=RandomizedSearchAlgorithm(Hyper\_parameters$)     $C\_metric=Evaluate(Features,\;C,\;Accuracy,\;F1_{score},Recall,\;Precision,\;ROC$)                   $List_{Classifier\_evaluation\_metrics}.append\;\left(C_{i},C\_metric\right)$  ***end for***10.Final classifier is obtained as: $Clf=ArgMax\left\{List_{Classifier\_evaluation\_metrics}\right\}$     argmax operator returns the classifier that maximizes the evaluation metric. 11. **END algorithm**

## 3. Experimentation

This section describes the experimental data, the hyperparameter tuning process for the various ML models, and the caries prediction model results. The initial stage in the experiment was to construct different subsets of features from the dataset using feature selection and feature importance methods (explained in Section 2.3 and in the Supplementary Material). Following that, the ML models were applied to the complete feature set and the different subsets of features to demonstrate the benefit of feature selection. Finally, the results of each model were compared to determine which model performed best.

### 3.1. Dataset

The 2018 pediatric oral health examination data were used to train, validate, and evaluate the proposed method’s effectiveness and performance. This dataset has 43 attributes in total. *Chi-Square*, *Relief F*, and *mRMR* techniques were utilized, with the number of features (k) adjusted to forty-three, forty, thirty-five, thirty, twenty-five, twenty, fifteen, ten, and five per experiment. The correlation method was also used to determine which variables to investigate further and to perform rapid hypothesis testing. A cutoff of 0.85 was set while performing the correlation technique, and features with a correlation value greater than that of the threshold were removed from the dataset’s 43 features. In addition, *GINI* feature importance was applied to these four methods (*Chi-square, Relief F*, *mRMR*, and *Correlation*). The dataset was subjected to eight different approaches. The different subset numbers of features to which the feature selection and importance techniques were applied are shown in Table S2 (see Supplementary File). Except for LSTM and SVM, the study used feature importance with the GBDT, RF, and LR models. Because SVM lacks feature importance properties, they cannot be applied [27]. For LSTM, only the permutation importance algorithm can be applied [28]. The dataset attribute descriptions are available in Table S1 (see Supplementary).

### 3.2. Hyperparameters of Different Machine Learning Models

A well-selected training dataset and properly tuned ML algorithms were necessary to accurately predict dental caries. In this study, five models were employed, with 80% of the data used for training and validation, while the remaining 20% were set aside for testing and determining the best model for caries prediction. The *hyperparameter* tuning was caried out through randomizedSearch instead of GridSearch. Table 1 shows the models used, the different feature selection and feature importance techniques, the *hyperparameters* of each model, and the optimal hyperparameter values achieved using the randomizedSearch algorithm.

### 3.3. Results

This section discusses the result obtained from the performed experimentation. The ML models were trained with the complete feature sets first and then with various reduced sets of features to demonstrate the effects of feature selection and importance techniques on the classification performances. Out of the 80% ratio of the dataset used for cross-validation training, 20% was utilized to evaluate the model efficacy, highlight selection bias or overfitting issues, and provide insights into how the model would generalize to an independent dataset. The results described below, which are the test data results, only present the excellent accuracy of each experimental condition.

#### 3.3.1. Performance of the Classifiers without Feature Selection

**Figure 3:**
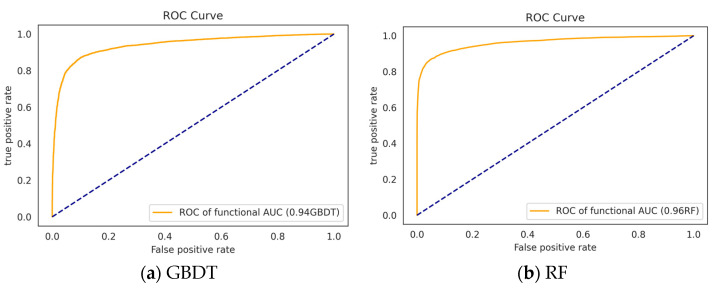

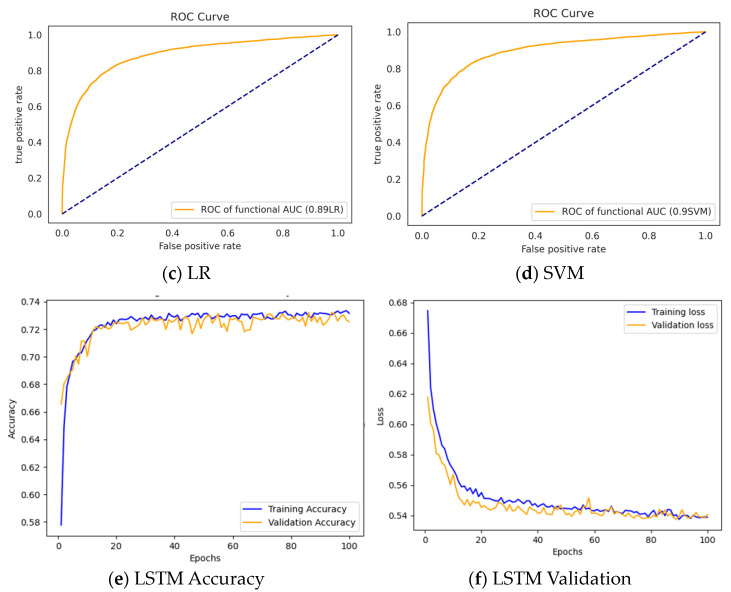
Evaluation of different models using (**a**–**d**) ROC curve of the classifiers trained using the full features. (**e**,**f**) Training vs. validation loss LSTM.

This subsection presents the experimental findings achieved when training the GBDT, RF, LR, SVM, and LSTM models with the full feature set. A summary of these observations is provided in Table 2. Further, Figure 3 depicts the AUC-ROC curves for the GBDT, RF, LR, and SVM algorithms, as well as the training vs. validation accuracy and loss for the LSTM model. The findings reveal that RF outperformed the other ML classifiers, with F1-score, precision, recall, and accuracy values of 0.87, 0.92, 0.86, and 0.91, respectively.

#### 3.3.2. Performance of the Classifiers after Feature Selection

**Figure 4:**
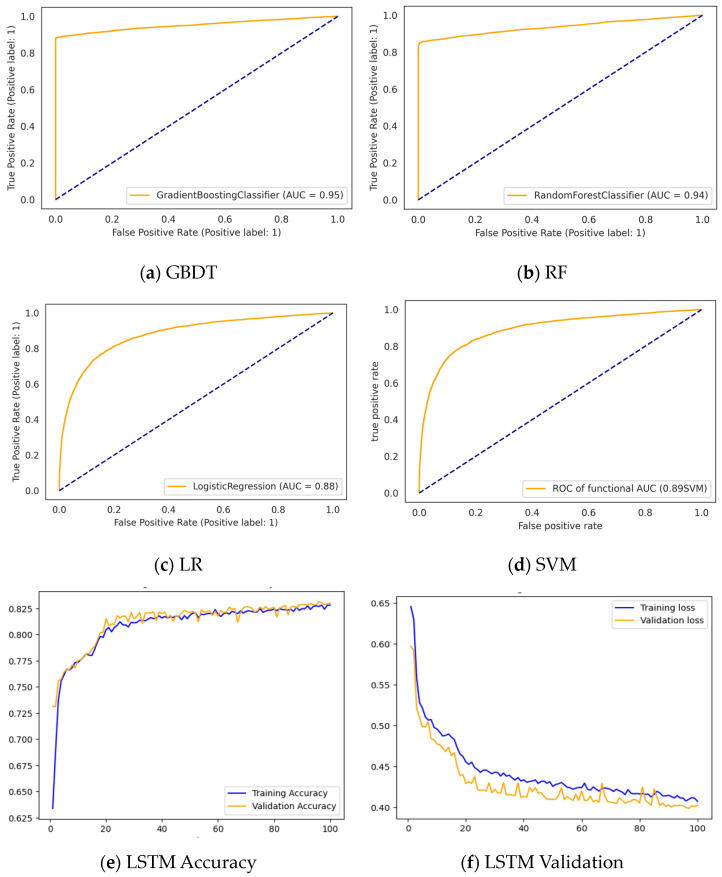
Evaluation of different models using (**a**–**d**) ROC curve of the classifiers trained using the subsets. (**e**,**f**) Training vs. validation loss LSTM.

Various reduced feature sets were produced in this study using the *Chi-Square*, *Relief F*, *mRMR*, *Correlation*, and *GINI* methods. The combination of the *mRMR* feature selection and *GINI* importance algorithms enabled the selection of features with the highest relevance to the label. The experimental results of the experimentation performed using various subsets of features are shown in Table 3 and Figure 4. Table 3 depicts the feature selection procedures (*mRMR + GINI*, *Relief F + GINI*, *Chi-Square + GINI*, *Relief F*, and *Chi-Square*) used to construct the datasets, with different numbers of features chosen depending on their importance in the prediction of the target variable.

The results obtained with the reduced feature sets were compared to show the efficacy of feature selection on model performance. As seen in Table 3, all the classifiers employed outperformed their respective performances when trained on the complete feature set (as shown in Table 2). The F1-score, precision, recall, accuracy, and AUC values of the proposed model (combining *mRMR + GINI* and GBDT) were 93.79%, 99.84%, 88.44%, 95.19%, and 95%, respectively, which outperformed the RF, LR, SVM, and LSTM models both before and after feature selection, as well as the conventional GBDT. This gain in performance highlights the usefulness of feature selection in the ML models. As a result, the proposed model is an effective method for predicting dental caries.

Table 3 summarizes the study’s findings and presents each model’s performance. The proposed approach demonstrated the best predictive performance. As shown in Table 3 and Table S2, each model’s appropriate number of features varied. Table 3 presents the best performance produced via various optimal feature sets. The accuracy of *mRMR + GINI* paired with GBDT was 95.19%, which was 5.53% higher than the traditional GBDT. RF (with *Relief F + GINI* for feature selection) produced an accuracy of 95.13%, 5.08% higher than the conventional RF; similarly, LSTM (with *Chi-Square + GINI*), SVM (with *Relief F*), and LSTM (with *Chi-Square*) achieved accuracies of 82.56%, 90.39%, and 84%, respectively. These results were 0.53%, 9.11%, and 9.33% higher than their counterparts trained on the complete feature sets, as shown in Table 2.

## 4. Discussion

Dental caries is rapidly increasing and recognized as a significant public health problem beyond personal health care. In addition, the prevention and early detection of dental caries are critical for reducing the social costs of dental caries that will occur in the future. Currently, a caries diagnosis is performed using radiographs or probes. According to the clinical experience of dental specialists, these procedures demonstrate significant variations in the accuracy and reliability of dental caries diagnoses. Given the difficulties and limitations of diagnoses based on the clinician’s subjective opinion and experience, research on and the development of ML-based dental caries decision support systems (DSSs) are required. These DSS tools will aid in the prevention of dental caries, the management of oral hygiene, the improvement of dietary-caries-related food habits, and the reduction of diagnostic time and expense.

The main purpose of this study was to construct a dataset comprising the most relevant features to enable an effective prediction of dental caries. Moreover, a model was proposed that can effectively and accurately classify dental caries using the selected feature set. Four ML algorithms (RF, LR, SVM, and GBDT) and one DL were trained on both the complete feature set and the optimized datasets (consisting of reduced feature sets).

The present study used the *Chi-Square*, *Relief F*, *mRMR*, *Correlation*, and *GINI* methods to obtain the optimal dataset. The most optimal dataset consisted of a feature set reduced to 18 out of 43 features, resulting in a 6% improvement in accuracy over training with the complete feature set. The experimental results reveal that the models trained on the reduced feature sets performed better than their respective counterparts trained on the complete feature sets. The proposed model trained on the reduced set of features remarkably predicted dental caries and yielded better performances than the previously published questionnaire-based dental caries prediction DSS.

Karhade, D. S. et al. [22] used data from 6404 people to study the classification of early childhood dental caries. Their proposed model yielded an AUC-ROC of 0.74, a sensitivity of 0.67, and a positive predictive value of 0.64. Park Y.H. et al. [24] performed a similar study in children under the age of 5 and reported an AUC-ROC value of 0.784 for LR, 0.785 for XGBoost, 0.780 for RF, and 0.780 for LightGBM. These findings outperformed those of Karhade, D. S. et al. [22] and Park, Y. H. et al. [24]. Our proposed prediction model’s AUC-ROC values were 0.96, 0.95, 0.89, 0.90, and 0.83 for RF, GBDT, LR, SVM, and LSTM, respectively. The current study’s data collection included 22,287 survey data samples. The proposed (*mRMR + GINI* and GBDT) model in our work achieved an accuracy of 95%, an F1-score of 93%, a precision of 99%, and a recall of 88%. However, because the data utilized were from the Korean child population, the performance analysis is only indicative of the Korean population rather than other groups. Simultaneously, an absolute comparison with other published studies was not possible due to the data’s privacy restrictions, resulting in its nonpublic availability.

## 5. Conclusions

The analysis of dental caries is one of the most frequent topics for modern-day oral health care research due to its severity and alarming rising rate. This study proposed an approach that combines *mRMR + GINI* feature selection and the GBDT classifier to improve the detection of dental caries. Five ML classifiers (LR, RF, SVM, LSTM, and GBDT) were implemented as benchmarks for a performance comparison. To start, the relevance of the various attributes was computed using *Chi-Square*, *Relief F*, *mRMR*, *Correlation*, and *GINI*. The classifiers were then trained using both the reduced and complete feature sets. The experimental results show that the feature selection improved the classifiers’ performance. The proposed approach achieved superior performance over the other classifiers and methods in the recent literature. Therefore, combining *mRMR + GINI* and GBDT is a practical approach to dental caries detection and can be potentially applied for the early detection of dental caries through DSS diagnosis tools.

In this experiment, the importance of features was selected through the *GINI* technique. The *GINI* technique employed to perform feature importance could not be used with the SVM and LSTM models and could only be used with LR, RF, and GBDT. In future work, we plan to use datasets collected from Korea and other countries to analyze similarities in dental caries occurrence. In addition, we intend to apply permutation importance (a feature importance technique) to improve the models’ performance. The proposed model shows promising performance and can be applied as a diagnostic aid to identify patients with dental caries. It can also make practical suggestions for caries prevention and treatment plan design. Through this, it is possible to drastically reduce patient diagnosis time and social costs due to tooth decay.

## Figures and Tables

**Table 1:** Optimizable parameters for different models.

Models	Feature Selection/ Feature Importance	Parameters	Optimal Values
GBDT	mRMR + GINI	subsample	0.80
		n_estimators	200
		min_samples_leaf	8
		max_features	5
		max_depth	320
		learning_rate	0.02
RF	Relief F + GINI	n_estimators	320
		min_samples_leaf	1
		max_features	5
		max_depth	None
LR	Chi-Square + GINI	solver	sag
		penalty	l2
		C	5
SVM	Relief F	probability	True
		kernel	rbf
		gamma	0.01
		C	10
LSTM	Chi-Square	learning rate	0.001
		beta_1	0.09
		beta_2	0.999
		epsilon	1 × 10^−2^
		epochs	100

**Table 2:** Classifier performance when trained with the complete set of features.

Models	#of Features	F1-Score	Precision	Recall	Accuracy
GBDT	43	0.8635	0.9490	0.7921	0.8966
RF		0.8868	0.9186	0.8572	0.9105
LR		0.7773	0.7959	0.7598	0.8203
SVM		0.7862	0.7434	0.8345	0.8128
LSTM		0.7575	0.7428	0.7436	0.7467

**Table 3:** Performance of the classifiers trained with the subset.

Models	Feature Selection	# of Features	F1-Score	Precision	Recall	Accuracy
GBDT	mRMR + GINI	18	0.9379	0.9984	0.8844	0.9519
RF	Relief F + GINI	20	0.9372	0.9978	0.8835	0.9513
LR	Chi-Square + GINI	40	0.7814	0.8012	0.7625	0.8256
SVM	Relief F	43	0.8806	0.9028	0.8596	0.9039
LSTM	Chi-Square	15	0.8300	0.8400	0.8300	0.8400

## Data Availability

Not applicable.

## Supplementary Materials

The following supporting information can be downloaded at: https://www.mdpi.com/article/10.3390/bioengineering10020245/s1, Table S1. Description of attributes of the survey dataset used in our experiment; Table S2. The performance of difference models used. References [29,30,31,32,33,34,35,36,37,38,39,40,41,42,43] are cited in the supplementary materials.

## References

[1] Institute for Health Metrics and Evaluation (IHME) Explore Results from the 2019 Global Burden of Disease (GBD) Study. https://vizhub.healthdata.org/gbd-results/.

[2] (2021). Health Insurance Review and Assessment Service HIRA. https://www.hira.or.kr/bbsDummy.do?pgmid=HIRAA020041000100&brdScnBltNo=4&brdBltNo=10368&pageIndex=1.

[3] Rimi I.F., Arif M.A.I., Akter S., Rahman M.R., Islam A.H.M.S., Habib M.T. (2022). Machine Learning Techniques for Dental Disease Prediction. Iran J. Comput. Sci..

[4] Zhang X., Liang Y., Li W., Liu C., Gu D., Sun W., Miao L. (2022). Development and Evaluation of Deep Learning for Screening Dental Caries from Oral Photographs. Oral Dis..

[5] Lee S., Oh S., Jo J., Kang S., Shin Y., Park J. (2021). Deep Learning for Early Dental Caries Detection in Bitewing Radiographs. Sci. Rep..

[6] Estai M., Tennant M., Gebauer D., Brostek A., Vignarajan J., Mehdizadeh M., Saha S. (2022). Evaluation of a Deep Learning System for Automatic Detection of Proximal Surface Dental Caries on Bitewing Radiographs. Oral Surg. Oral Med. Oral Pathol. Oral Radiol..

[7] Lee J.-H., Kim D.-H., Jeong S.-N., Choi S.-H. (2018). Detection and Diagnosis of Dental Caries Using a Deep Learning-Based Convolutional Neural Network Algorithm. J. Dent..

[8] Megalan Leo L., Kalpalatha Reddy T. (2020). Dental Caries Classification System Using Deep Learning Based Convolutional Neural Network. J. Comput. Theor. Nanosci..

[9] Karhade D.S., Roach J., Shrestha P., Simancas-Pallares M.A., Ginnis J., Burk Z.J., Ribeiro A.A., Cho H., Wu D., Divaris K. (2021). An Automated Machine Learning Classifier for Early Childhood Caries. Pediatr. Dent..

[10] Ramos-Gomez F., Marcus M., Maida C.A., Wang Y., Kinsler J.J., Xiong D., Lee S.Y., Hays R.D., Shen J., Crall J.J. (2021). Using a Machine Learning Algorithm to Predict the Likelihood of Presence of Dental Caries among Children Aged 2 to 7. Dent. J..

[11] Park Y.-H., Kim S.-H., Choi Y.-Y. (2021). Prediction Models of Early Childhood Caries Based on Machine Learning Algorithms. Int. J. Environ. Res. Public Health.

[12] Remeseiro B., Bolon-Canedo V. (2019). A review of feature selection methods in medical applications. Comput. Biol. Med..

[13] Memiş S., Enginoğlu S., Erkan U. (2022). Fuzzy parameterized fuzzy soft k-nearest neighbor classifier. Neurocomputing.

[14] Saberi-Movahed F., Rostami M., Berahmand K., Karami S., Tiwari P., Oussalah M., Band S.S. (2022). Dual regularized unsupervised feature selection based on matrix factorization and minimum redundancy with application in gene selection. Knowl.-Based Syst..

[15] Azadifar S., Rostami M., Berahmand K., Moradi P., Oussalah M. (2022). Graph-based relevancy-redundancy gene selection method for cancer diagnosis. Comput. Biol. Med..

[16] Singh D., Singh B. (2020). Investigating the Impact of Data Normalization on Classification Performance. Appl. Soft Comput..

[17] Chawla N.V., Bowyer K.W., Hall L.O., Kegelmeyer W.P. (2002). SMOTE: Synthetic Minority Over-Sampling Technique. J. Artif. Intell. Res..

[18] Memiş S., Enginoğlu S., Erkan U. (2022). A classification method in machine learning based on soft decision-making via fuzzy parameterized fuzzy soft matrices. Soft Comput..

[19] Hu Q., Si X.-S., Qin A.-S., Lv Y.-R., Zhang Q.-H. (2020). Machinery Fault Diagnosis Scheme Using Redefined Dimensionless Indicators and MRMR Feature Selection. IEEE Access.

[20] Bugata P., Drotar P. (2020). On Some Aspects of Minimum Redundancy Maximum Relevance Feature Selection. Sci. China Inf. Sci..

[21] Wang Y., Li X., Ruiz R. (2022). Feature Selection with Maximal Relevance and Minimal Supervised Redundancy. IEEE Trans. Cybern..

[22] Ghasemi F., Neysiani B.S., Nematbakhsh N. Feature Selection in Pre-Diagnosis Heart Coronary Artery Disease Detection: A Heuristic Approach for Feature Selection Based on Information Gain Ratio and Gini Index. Proceedings of the 2020 6th International Conference on Web Research (ICWR).

[23] Sung S.-H., Kim S., Park B.-K., Kang D.-Y., Sul S., Jeong J., Kim S.-P. (2021). A Study on Facial Expression Change Detection Using Machine Learning Methods with Feature Selection Technique. Mathematics.

[24] Zhang W., Yu J., Zhao A., Zhou X. (2021). Predictive Model of Cooling Load for Ice Storage Air-Conditioning System by Using GBDT. Energy Rep..

[25] Liang W., Luo S., Zhao G., Wu H. (2020). Predicting Hard Rock Pillar Stability Using GBDT, XGBoost, and LightGBM Algorithms. Mathematics.

[26] Li S., Lin Y., Zhu T., Fan M., Xu S., Qiu W., Chen C., Li L., Wang Y., Yan J. (2021). Development and external evaluation of predictions models for mortality of COVID-19 patients using machine learning method. Neural Comput. Appl..

[27] SVC’ Object Has No Attribute “Feature_importances_”. https://stackoverflow.com/questions/59681421/svc-object-has-no-attribute-feature-importances.

[28] Spencer R., Thabtah F., Abdelhamid N., Thompson M. (2020). Exploring Feature Selection and Classification Methods for Predicting Heart Disease. Digit. Health.

[29] Scikit-Learn Developers Sklearn Feature Selection Chi. https://scikit-learn.org/stable/modules/generated/sklearn.feature_selection.chi2.html#sklearn.feature_selection.chi2.

[30] Thaseen I.S., Kumar C.A., Ahmad A. (2019). Integrated intrusion detection model using chi-square feature selection and ensemble of classifiers. Arab. J. Sci. Eng..

[31] Zhang B., Li Y., Chai Z. (2022). A Novel Random Multi-Subspace Based ReliefF for Feature Selection. Knowl.-Based Syst..

[32] Ghosh P., Azam S., Jonkman M., Karim A., Shamrat F.M.J.M., Ignatious E., Shultana S., Beeravolu A.R., De Boer F. (2021). Efficient Prediction of Cardiovascular Disease Using Machine Learning Algorithms with Relief and LASSO Feature Selection Techniques. IEEE Access.

[33] Zhou H., Zhang J., Zhou Y., Guo X., Ma Y. (2021). A Feature Selection Algorithm of Decision Tree Based on Feature Weight. Expert Syst. Appl..

[34] Saidi R., Bouaguel W., Essoussi N., Hassanien A.E. (2019). Hybrid Feature Selection Method Based on the Genetic Algorithm and Pearson Correlation Coefficient. Machine Learning Paradigms: Theory and Application.

[35] Kumar M.S., Soundarya V., Kavitha S., Keerthika E.S., Aswini E. Credit Card Fraud Detection Using Random Forest Algorithm. Proceedings of the 3rd International Conference on Computing and Communications Technologies (ICCCT).

[36] Xing J., Wang H., Luo K. (2019). Predictive single-step kinetic model of biomass devolatilization for CFD applications: A comparison study of empirical correlations (EC), artificial neural networks (ANN) and random forest (RF). Renew. Energy.

[37] Otchere D.A., Arbi Ganat T.O., Gholami R., Ridha S. (2021). Application of Supervised Machine Learning Paradigms in the Prediction of Petroleum Reservoir Properties: Comparative Analysis of ANN and SVM Models. J. Pet. Sci. Eng..

[38] Iwendi C., Mahboob K., Khalid Z., Javed A.R., Rizwan M., Ghosh U. (2022). Classification of COVID-19 Individuals Using Adaptive Neuro-Fuzzy Inference System. Multimed. Syst..

[39] Chen H., Zhang C., Jia N., Duncan I., Yang S., Yang Y. (2021). A machine learning model for predicting the minimum miscibility pressure of CO_2_ and crude oil system based on a support vector machine algorithm approach. Fuel.

[40] Kurt I., Ture M., Kurum A.T. (2008). Comparing performances of logistic regression, classification and regression tree, and neural networks for predicting coronary artery disease. Expert Syst. Appl..

[41] Hochreiter S., Schmidhuber J. (1997). Long Short-Term Memory. Neural Comput..

[42] Hamayel M.J., Owda A.Y. (2021). A Novel Cryptocurrency Price Prediction Model Using GRU, LSTM and Bi-LSTM Machine Learning Algorithms. AI.

[43] Absar N., Uddin M.N., Khandaker M.U., Ullah M.H. (2022). The efficacy of deep learning based LSTM model in forecasting the outbreak of contagious diseases. Infect. Dis. Model..

