# Supplementary material for: Optimal Feature Selection-Based Dental Caries Prediction Model Using Machine Learning for Decision Support System
Source: Bioengineering (Basel). 2023 Feb 13;10(2):245. doi: 10.3390/bioengineering10020245 (PMC9952690; doi:10.3390/bioengineering10020245)
Supplement: Supplementary file 1 [file bioengineering-10-00245-s001.zip › bioengineering-2182751-supplementary.pdf]

Table S1. Description of attributes of the survey dataset used in our experiment.

**Variable Name**	**Variable Description**
act_caries	Presence of dental caries (label)
Sido_No	Area of residence of the subject of dental examination
Region_No	Region of residence of the subject of dental examination
Gender	Gender
Prev_caries	Previously experienced dental caries
X1	Awareness of dental and gum oral health
X2	Dental treatment experience in the past year
X3	Experience of needing dental treatment but not receiving treatment
X4_1	Teeth brushed before breakfast
X4_2	Teeth brushed after breakfast
X4_3	Teeth brushed before lunch
X4_4	Teeth brushed after lunch
X4_5	Teeth brushed before dinner
X4_6	Teeth brushed after dinner
X4_7	Teeth brushed after snack
X4_8	Teeth brushed before going to bed
X4_9	Teeth not being brushed
X5_1	Regular dental floss usage Frequency
X5_2	Handle floss usage Frequency
X5_3	Mouth wash usage Frequency
X5_4	Electric toothbrush usage Frequency
X5_5	Oral care product usage?
X6	Use of toothpaste
X7	Use of fluoride toothpaste
X8	Sticky snacks eaten today?
X9	Sticky snacks eaten yesterday?
X10	Pain in the gums or bleeding when brushing
X11	Pain or discomfort in your teeth / past 1 year
X12	Parents smoking
X13	Smoking experience
X14_1	Living with grandfather
X14_2	Living with grandmother
X14_3	Living with father
X14_4	Living with stepfather
X14_5	Living with mother
X14_6	Living with stepmother
X14_7	Living with older brother / older sister
X14_8	Living with younger brother / younger sister
X14_9	Not living with any of the above family member (orphans included)
X15_1	Household economic status
X16	Weekly allowance
Calculus	Have tartar buildup
Bleeding	Gingival bleeding
Fluorosis	Tooth speckle

Table S2. The performance of difference models used.

Models	Setting Features	Full Features					Feature Selection						Feature Importance					
		#of Features	F1-score	Precision	Recall	Accuracy	Method	#of Features	F1-score	Precision	Recall	Accuracy	Method	#of Features	F1-score	Precision	Recall	Accuracy
GBDT	43	43	0.8635	0.9490	0.7921	0.8966	Chi-Square	43	0.9358	0.9994	0.8799	0.9503	Chi-Square + GINI	16	0.9374	0.9984	0.8835	0.9515
RF			0.8868	0.9186	0.8572	0.9105			0.9342	0.9994	0.8771	0.9491		20	0.9370	0.9982	0.8830	0.9512
LR			0.7773	0.7959	0.7598	0.8203			0.7754	0.7996	0.7530	0.8202		40	0.7814	0.8012	0.7625	0.8256
SVM			0.7862	0.7434	0.8345	0.8128			0.8804	0.9021	0.8599	0.9037		N/A				
LSTM			0.7575	0.7428	0.7436	0.7467			0.8300	0.8300	0.8300	0.8400						
GBDT	N/A						Relief F		0.9358	0.9990	0.8802	0.9503	Relief F + GINI	17	0.9360	0.9937	0.8847	0.9504
RF									0.9342	0.9994	0.8771	0.9491		20	0.9372	0.9978	0.8835	0.9513
LR									0.7767	0.7960	0.7586	0.8202		41	0.7805	0.7622	0.7622	0.8239
SVM									0.8806	0.9028	0.8596	0.9039		N/A				
LSTM									0.8300	0.8400	0.8300	0.8400						
GBDT							mRMR		0.9356	0.9987	0.8792	0.9501	mRMR + GINI	20	0.9378	0.9990	0.8837	0.9518
RF									0.8844	0.9185	0.8530	0.9081		21	0.8785	0.88979	0.8598	0.9023
LR									0.7762	0.7986	0.7552	0.8205		41	0.7814	0.8012	0.7625	0.8247
SVM									0.8800	0.8979	0.8629	0.9030		N/A				
LSTM									0.8300	0.8400	0.8200	0.8400						
GBDT							Correlation	42	0.9355	0.9994	0.8793	0.9500	Correlation + GINI	17	0.9375	0.9964	0.8852	0.9515
RF									0.8893	0.9232	0.8580	0.9120		21	0.8814	0.9009	0.8628	0.9046
LR									0.7749	0.7985	0.7529	0.8198		42	0.7813	0.8012	0.7623	0.8246
SVM									0.8831	0.9032	0.8640	0.9057		N/A				
LSTM									0.8300	0.8400	0.8200	0.8400						
GBDT	40	N/A					Chi-Square	40	0.9358	0.9998	0.8796	0.9503	Chi-Square + GINI	16	0.9367	0.9990	0.8818	0.9510
RF									0.9342	0.9997	0.8769	0.9491		18	0.9359	0.9956	0.8830	0.9503
LR									0.7675	0.7888	0.7477	0.8135		39	0.7703	0.7882	0.7531	0.8154
SVM									0.8549	0.8667	0.8434	0.8819		N/A				
LSTM									0.8300	0.8300	0.8200	0.8400						
GBDT	40	N/A					Relief F	40	0.9355	0.9989	0.8797	0.9500	Relief F + GINI	15	0.9356	0.9914	0.8857	0.9499
RF									0.9342	0.9989	0.8775	0.9491		18	0.9353	0.9933	0.8837	0.9498
LR									0.7740	0.7959	0.7535	0.8186		38	0.7788	0.7985	0.7601	0.8226
SVM									0.8157	0.8127	0.8187	0.8480		N/A				
LSTM									0.8300	0.8300	0.8200	0.8400						
GBDT							mRMR		0.9355	0.9989	0.8798	0.9500	mRMR	15	0.9370	0.9968	0.8840	0.9512

Models	Setting Features	Full Features #of Features	F1-score	Precision	Recall	Accuracy	Feature Selection Method	#of Features	F1-score	Precision	Recall	Accuracy
RF									0.8706	0.8935	0.8489	0.8959
LR									0.7571	0.7758	0.7395	0.8044
SVM									0.8474	0.8540	0.8410	0.8751
LSTM									0.8100	0.8100	0.8000	0.8100
GBDT							Correlation	42	0.9355	0.9994	0.8793	0.9500
RF									0.8893	0.9232	0.8580	0.9120
LR									0.7709	0.7942	0.7491	0.8164
SVM									0.7881	0.8145	0.7634	0.8307
LSTM									0.8300	0.8300	0.8300	0.8400
GBDT	35	N/A					Chi-Square	35	0.9351	0.9988	0.8791	0.9497
RF									0.9331	0.9984	0.8759	0.9482
LR									0.7197	0.7597	0.6838	0.7804
SVM									0.8190	0.8126	0.8256	0.8496
LSTM									0.8300	0.8300	0.8300	0.8400
GBDT							Relief F		0.9333	0.9994	0.8756	0.9484
RF									0.9264	0.9821	0.8767	0.9425
LR									0.7381	0.7700	0.7089	0.7926
SVM									0.7389	0.7875	0.6960	0.7971
LSTM									0.8000	0.8000	0.7900	0.8100
GBDT							mRMR		0.9363	0.9996	0.8807	0.9506
RF									0.8502	0.8614	0.8394	0.8781
LR									0.7184	0.7337	0.7039	0.7726
SVM									0.7740	0.7364	0.8157	0.8036
LSTM									0.7800	0.7800	0.7800	0.7900
GBDT							Correlation	42	0.9355	0.9994	0.8793	0.9500
RF	35	N/A						42	0.8893	0.9232	0.8580	0.9120
LR									0.7748	0.7983	0.7528	0.8196
SVM									0.7525	0.8097	0.7572	0.8265
LSTM									0.8300	0.8300	0.8300	0.8400
GBDT	30	N/A					Chi-Square	30	0.9345	0.9992	0.8778	0.9493
RF									0.9325	0.9945	0.8778	0.9476
LR									0.7058	0.7485	0.6680	0.7706

Models	Feature Importance Method	#of Features	F1-score	Precision	Recall	Accuracy
RF	+ GINI	24	0.8619	0.8465	0.8779	0.8886
LR		38	0.7648	0.7814	0.7490	0.8108
SVM		N/A				
LSTM						
GBDT	Correlation + GINI	17	0.9357	0.9937	0.8842	0.9501
RF		21	0.8795	0.8996	0.86024	0.9031
LR		41	0.7814	0.8012	0.7625	0.8247
SVM		N/A				
LSTM						
GBDT	Chi-Square + GINI	16	0.9351	0.9958	0.8814	0.9498
RF		18	0.9355	0.9954	0.8824	0.9500
LR		33	0.7302	0.7596	0.7031	0.7866
SVM		N/A				
LSTM						
GBDT	Relief F + GINI	13	0.9321	0.9966	0.8755	0.9476
RF		17	0.9284	0.9799	0.8821	0.9441
LR		33	0.7296	0.7687	0.6944	0.7886
SVM		N/A				
LSTM						
GBDT	mRMR + GINI	15	0.9370	0.9962	0.8844	0.9511
RF		21	0.8480	0.8576	0.8386	0.8765
LR		33	0.7249	0.7384	0.7119	0.7780
SVM		N/A				
LSTM						
GBDT	Correlation+ GINI	14	0.9334	0.9891	0.8837	0.9482
RF		21	0.8757	0.8968	0.8555	0.9002
LR		41	0.7814	0.8012	0.7625	0.8247
SVM		N/A				
LSTM						
GBDT	Chi-Square + GINI	12	0.9361	0.9958	0.8831	0.9505
RF		16	0.9321	0.9888	0.8816	0.9473
LR		28	0.7104	0.7491	0.6754	0.7737

Models	Setting Features	Full Features					Feature Selection						Feature Importance					
		#of Features	F1-score	Precision	Recall	Accuracy	Method	#of Features	F1-score	Precision	Recall	Accuracy	Method	#of Features	F1-score	Precision	Recall	Accuracy
SVM									0.7929	0.7769	0.8096	0.8256		N/A				
LSTM									0.7900	0.7900	0.7900	0.8000						
GBDT							Relief F		0.9227	0.9997	0.8568	0.9408	Relief F + GINI	13	0.9168	1.0000	0.8465	0.9369
RF									0.9108	0.9953	0.8397	0.9322		15	0.9062	0.9856	0.8386	0.9287
LR									0.6767	0.7657	0.6063	0.7611		28	0.6897	0.7679	0.6259	0.7686
SVM									0.6851	0.7684	0.6181	0.7656		N/A				
LSTM									0.7600	0.7800	0.7500	0.7800						
GBDT							mRMR		0.9359	0.9998	0.8798	0.9503	mRMR + GINI	18	0.9379	0.9984	0.8844	0.9519
RF									0.8335	0.8409	0.8265	0.8639		19	0.8299	0.88356	0.8244	0.8612
LR									0.7127	0.7275	0.6986	0.7678		39	0.7081	0.7312	0.6864	0.7675
SVM									0.7155	0.7186	0.7125	0.7663		N/A				
LSTM									0.7800	0.7800	0.7800	0.7900						
GBDT							Correlation	42	0.9355	0.9994	0.8793	0.9500	Correlation + GINI	14	0.9311	0.9852	0.8826	0.9463
RF									0.8893	0.9232	0.8580	0.9120		21	0.8757	0.8968	0.8555	0.9002
LR									0.7748	0.7983	0.7528	0.8196		41	0.7814	0.8012	0.7625	0.8247
SVM									0.7841	0.8108	0.7591	0.8276		N/A				
LSTM									0.8400	0.8200	0.8300	0.8400						
GBDT	25	N/A					Chi-Square	25	0.9337	0.9981	0.8772	0.9486	Chi-Square + GINI	12	0.9328	0.9974	0.8760	0.9481
RF									0.9247	0.9779	0.8771	0.9412		14	0.9249	0.9689	0.8847	0.9410
LR									0.6844	0.7400	0.6369	0.7579		23	0.6847	0.7380	0.6386	0.7584
SVM									0.7565	0.7916	0.7245	0.8077		N/A				
LSTM									0.8200	0.8300	0.8300	0.8300						
GBDT							Relief F		0.8850	0.9991	0.7944	0.9419		12	0.8630	0.9995	0.7594	0.9010
RF									0.8428	0.9948	0.7312	0.8875		14	0.8489	0.9923	0.7416	0.8915
LR	25	N/A					Relief F	25	0.6236	0.5983	0.6512	0.6759	Relief F + GINI	24	0.6333	0.6056	0.6638	0.6843
SVM									0.6329	0.5893	0.6841	0.6729		N/A				
LSTM									0.8000	0.7900	0.7900	0.8000						
GBDT							mRMR		0.9344	0.9972	0.8792	0.9492	mRMR + GINI	13	0.9327	0.9877	0.8835	0.9476
RF									0.8161	0.8221	0.8104	0.8494		18	0.8102	0.8126	0.8077	0.8445
LR									0.6943	0.7141	0.6757	0.7548		24	0.7095	0.7238	0.6958	0.7659
SVM									0.7572	0.8872	0.6605	0.8253		N/A				
LSTM									0.8300	0.8300	0.8300	0.8300						

Models	Setting Features	Full Features					Feature Selection						Feature Importance					
		#of Features	F1-score	Precision	Recall	Accuracy	Method	#of Features	F1-score	Precision	Recall	Accuracy	Method	#of Features	F1-score	Precision	Recall	Accuracy
GBDT							Correlation	42	0.9355	0.9994	0.8793	0.9500	Correlation + GINI	13	0.9271	0.9770	0.8821	0.9630
RF									0.8893	0.9232	0.8580	0.9120		21	0.8757	0.8968	0.8555	0.9002
LR									0.7748	0.7983	0.7528	0.8196		41	0.7814	0.8012	0.7625	0.8247
SVM									0.7659	0.7913	0.7422	0.8137		N/A				
LSTM									0.8300	0.8200	0.8300	0.8400						
GBDT	20	N/A					Chi-Square	20	0.9282	0.9989	0.8670	0.9447	Chi-Square + GINI	10	0.9265	0.9969	0.8654	0.9436
RF									0.9419	0.9757	0.8687	0.9369		11	0.9134	0.9635	0.8682	0.9323
LR									0.6738	0.7233	0.6309	0.7482		19	0.6684	0.7158	0.6269	0.7158
SVM									0.7027	0.7175	0.6888	0.7598		N/A				
LSTM									0.8200	0.8200	0.8200	0.8300						
GBDT							Relief F		0.7511	0.9996	0.6017	0.8355	Relief F + GINI	11	0.7283	1.0	0.5727	0.8244
RF									0.7090	0.9997	0.5494	0.8140		10	0.6907	0.9983	0.5280	0.8057
LR									0.3079	0.68332	0.1988	0.6315		19	0.3002	0.6719	0.1932	0.6298
SVM									0.6508	0.5448	0.8082	0.6424		N/A				
LSTM									0.7900	0.8000	0.7800	0.8000						
GBDT							mRMR		0.9340	0.9994	0.8768	0.9490	mRMR + GINI	11	0.9338	0.9986	0.8769	0.9489
RF									0.7486	0.7849	0.7889	0.8238		18	0.794	0.7489	0.7990	0.8299
LR									0.6785	0.6968	0.6611	0.7416		19	0.6791	0.7025	0.6572	0.7448
SVM									0.7305	0.6978	0.7666	0.7667		N/A				
LSTM									0.7800	0.7900	0.7800	0.7900						
GBDT							Correlation	42	0.9355	0.9994	0.8793	0.9500	Correlation + GINI	11	0.9238	0.9725	0.8798	0.9404
RF									0.8893	0.9232	0.8580	0.9120		21	0.8787	0.8968	0.8555	0.9002
LR									0.7748	0.7983	0.7528	0.8196		41	0.7814	0.8012	0.7625	0.8247
SVM		N/A							0.7878	0.8118	0.7651	0.8300		N/A				
LSTM									0.8300	0.8400	0.8300	0.8400						
GBDT	15	N/A					Chi-Square	15	0.9152	0.9960	0.8466	0.8353	Chi-Square+ GINI	8	0.9164	0.9961	0.8486	0.9364
RF									0.8997	0.9990	0.8185	0.9248		9	0.9043	0.9939	0.8294	0.9278
LR									0.6330	0.7038	0.5754	0.7249		14	0.6422	0.7147	0.5831	0.7331
SVM									0.6385	0.7076	0.5818	0.7284		N/A				
LSTM									0.8300	0.8400	0.8300	0.8400						
GBDT							Relief F		0.5772	0.9997	0.4058	0.7549	Relief F + GINI	9	0.5660	1.0	0.3947	0.7513
RF									0.5763	0.9991	0.4051	0.7545		8	0.5278	1.0	0.3585	0.7365

Models	Setting Features	Full Features #of Features	F1-score	Precision	Recall	Accuracy	Feature Selection Method	#of Features	F1-score	Precision	Recall	Accuracy	Feature Importance Method	#of Features	F1-score	Precision	Recall	Accuracy
LR									0.0286	0.6159	0.0146	0.5899		14	0.0224	0.5322	0.0114	0.5897
SVM									0.0303	0.6085	0.0155	0.5899		N/A				
LSTM									0.7200	0.7300	0.7100	0.7300						
GBDT									0.9301	0.9941	0.8740	0.9459		10	0.9294	0.9938	0.8729	0.9455
RF									0.6943	0.7087	0.6807	0.7529		14	0.7020	0.7131	0.6912	0.7589
LR							mRMR		0.6516	0.6815	0.6244	0.7247	mRMR + GINI	14	0.6413	0.6776	0.6087	0.7202
SVM									0.6846	0.6621	0.7087	0.7307		N/A				
LSTM									0.7400	0.7400	0.7400	0.7500						
GBDT									0.9355	0.9994	0.8793	0.9500		9	0.9235	0.9852	0.8691	0.9408
RF									0.8893	0.9232	0.8580	0.9120		21	0.8757	0.8968	0.8555	0.9002
LR							Correlation	42	0.7748	0.7983	0.7528	0.8196	Correlation + GINI	41	0.7814	0.8012	0.7625	0.8247
SVM									0.8635	0.8972	0.8323	0.8915		N/A				
LSTM									0.8300	0.8300	0.8300	0.8400						
GBDT									0.8908	0.99827	0.80442	0.9187		6	0.8859	0.9995	0.7955	0.9158
RF									0.8517	0.9996	0.7419	0.8934		7	0.8613	1.0000	0.74564	0.8999
LR							Chi-Square		0.5407	0.6557	0.4603	0.6776	Chi-Square + GINI	9	0.5508	0.6615	0.4719	0.6838
SVM									0.5829	0.6464	0.5311	0.6867		N/A				
LSTM	10	N/A						10	0.6700	0.6900	0.6600	0.7000						
GBDT									0.2977	1.0000	0.1750	0.6597		8	0.2959	1.0000	0.1736	0.6605
RF									0.3023	1.0000	0.1782	0.6612	Relief F + GINI	8	0.2989	1.0000	0.1745	0.6613
LR							Relief F		0.0318	0.6017	0.0163	0.5898		9	0.0277	0.5157	0.0142	0.5895
SVM									0.0336	0.5934	0.0173	0.5899		N/A				
LSTM									0.3800	0.5400	0.5000	0.5900		N/A				
GBDT									0.9198	1.0000	0.8517	0.9388		8	0.9204	1.0000	0.8525	0.9394
RF									0.5927	0.6295	0.5601	0.6825		9	0.6082	0.6357	0.5830	0.6914
LR							mRMR	10	0.5731	0.6300	0.5258	0.6769	mRMR + GINI	9	0.5791	0.6284	0.5369	0.6793
SVM									0.6016	0.6302	0.5755	0.6856		N/A				
LSTM	10	N/A							0.6700	0.6800	0.6700	0.6900						
GBDT									0.9355	0.9994	0.8793	0.9500		9	0.9235	0.9852	0.8691	0.9408
RF									0.8893	0.9232	0.8580	0.9120	Correlation + GINI	21	0.8757	0.8968	0.8555	0.9002
LR							Correlation	42	0.7748	0.7983	0.7528	0.8196		41	0.7814	0.8012	0.7625	0.8247
SVM									0.7934	0.7483	0.8443	0.8194		N/A				

Models	Setting Features	Full Features					Feature Selection						Feature Importance					
		#of Features	F1-score	Precision	Recall	Accuracy	Method	#of Features	F1-score	Precision	Recall	Accuracy	Method	#of Features	F1-score	Precision	Recall	Accuracy
LSTM									0.8300	0.8400	0.8300	0.8400						
GBDT	5	N/A					Chi-Square	5	0.7532	0.9981	0.6049	0.8366	Chi-Square + GINI	3	0.7610	1.0000	0.6142	0.8415
RF									0.7455	0.9980	0.5952	0.8326		3	0.7555	1.0000	0.6071	0.8386
LR									0.4618	0.7227	0.3394	0.6738		4	0.4552	0.7185	0.3332	0.6724
SVM									0.4549	0.7186	0.3328	0.6711		N/A				
LSTM									0.6100	0.6900	0.6200	0.6700						
GBDT							Relief F		0.0884	1.0000	0.0462	0.6066	Relief F + GINI	3	0.0909	1.0000	0.0476	0.6087
RF									0.0828	1.0000	0.0432	0.6054		4	0.0828	1.0000	0.0432	0.6054
LR									0.0317	0.5530	0.0163	0.5888		4	0.0280	0.4797	0.0144	0.5886
SVM									0.0340	0.5575	0.1757	0.5891		N/A				
LSTM									0.6100	0.6100	0.6100	0.6300						
GBDT							mRMR		0.8346	1.0000	0.7163	0.8830	mRMR + GINI	4	0.8411	1.0000	0.7258	0.8873
RF									0.6125	0.5524	0.6874	0.6413		4	0.6007	0.5402	0.6765	0.6306
LR									0.5594	0.5506	0.5686	0.6305		4	0.5539	0.5416	0.5668	0.6249
SVM									0.6128	0.5492	0.6927	0.6388		N/A				
LSTM									0.8200	0.8300	0.8200	0.8300						
GBDT							Correlation	42	0.9355	0.9994	0.8793	0.9500	Correlation + GINI	9	0.9235	0.9852	0.8691	0.9408
RF									0.8893	0.9232	0.8580	0.9120		21	0.8757	0.8968	0.8555	0.9002
LR									0.7748	0.7983	0.7528	0.8196		41	0.7814	0.8012	0.7625	0.8247
SVM									0.7961	0.7509	0.8470	0.8217		N/A				
LSTM									0.8200	0.8300	0.8100	0.8300						

It can be observed that when feature selection and feature significance are used combined, the model's accuracy improves even when fewer features are employed than when feature selection alone is used. As stated in the paper, SVM and LSTM models are trained without using feature significance, hence the values in the table above are omitted.

1. Feature Selection

1.1. Chi-Square

The CHI statistic [29,30] calculates the degree of independence between the feature 'a_i' and the class label 'y_j' and compares it to the CHI distribution with degree of freedom set to *1*. As a result, the chi-square statistic is defined as follows:

Definition S1.

$$x^2\left(a_i, y_j\right) = \frac{N \cdot (TZ - YX)^2}{(T+X)(T+Z)(X+Z)(Y+Z)} \tag{10}$$

where T denotes the frequency of the feature 'a_i' and the class label 'y_j' in the dataset. X is the frequency with which 'a_i' appears without 'y_j'. Y is the frequency with which 'y_j' appears without 'a_i'. Z is the frequency with which neither 'y_j' nor 'a_i' appear in the sample. N represents the total number of records I = 1... 41 characteristics j = 1, 1 (class labels) [30].

1.2. Relief F

The Relief F algorithm does not restrict data types as a filter-based feature selection. Effective handling of nominal or continuity features, missing data, and noise tolerance [31]. This algorithm distinguishes whether the classifications are strongly or weakly correlated. If the classifications are strongly correlated, treat them as similar samples and keep those samples close together. On the contrary, samples with weakly correlated classifications are kept away. The feature weights are calculated by computing the nearest neighbor samples' within-class and between-class distances. This operation is repeated in order to update the weight vectors of features, and the weights of all features are eventually yielded [32].

The formula used in updating the weight value of features by the Relief F algorithm is given as, [33]

Definition S2.

$$W[A] = W[A_0] - \frac{\sum_{j=1}^{k} diff(A,\ x_i,\ H)}{mk} + \sum_{C \neq class(x_i)} \frac{p(C)}{1-p(class(x_i))} \cdot \frac{\sum_{j=1}^{k} diff(A,\ x_i,\ M(C))}{mk} \tag{11}$$

where, the weight coefficient determined at A_0 is $W[A]$. The original dataset feature set is represented by $W[A_0]$. x_i is sample, and H is the sample of the closest class to which x_I belongs. The difference between x_I and H for each attribute of A is represented by the formula $diff(A,\ x_I,\ H)$. The Manhattan distance between the values of the features is calculated by the $diff(A,\ x_I,\ H)$ A for two boundary conditions x_i and H, where k is the number of closest neighbors and m is the total number of iterations. The ratio of sample C to all samples is known as $p(C)$. The percentage of samples in the class to which sample x_i belongs to the entire sample is expressed as $p(class(x_i))$ The difference between x_i and $M(C)$ for each feature of A is represented by the expression $diff(A,\ x_i,\ M(C))$.

1.3. Correlation

Correlation analysis is a method that analyzes the linear relationship between two variables measured as curb variables. It analyzes whether variable *B* increases or decreases as variable *A* increases. Correlation analysis has various analysis methods, such as Pearson correlation analysis and Spearman correlation analysis, and this study conducted experiments using Pearson correlation analysis. The closer the coefficient is to *1*, the more significant the correlation, and the closer to *-1*, the inversely proportional. Each coefficient has a value of *+1* if it is precisely the same, *0* if it is completely different, and *-1* if it is precisely the same in the opposite direction [34].

2. Prediction Models.

2.1. RF (Random Forest)

RF is another name for Random Decision Forest (RDF), and it is used for classification, regression, and other tasks that require the construction of multiple decision trees. This RF Algorithm is based on supervised learning, and it has the advantage of being used for both classification and regression. The RF Algorithm outperforms all other existing systems in terms of accuracy, and it is the most widely used algorithm [35].

Definition S3.

$$MSE_{OOB} = \frac{\sum_{i-1}^{N_{tree}} (y_i - y_i^{OOB})^2}{N_{tree}} \tag{12}$$

$$R_{RF}^2 = 1 - \frac{MSE_{OOB}}{\sigma_y^2} \tag{13}$$

Where y_i and y_i^{OOB} are the actual and expected values from the OOB data. R_{RF}^2 and σ_y^2 are the coefficient of determination and variance of the predicted value of the OOB data respectively. The random forest output is the mean prediction (regression) or mean of the classes (classification) of the individual trees [36].

2.3. SVM (Support Vector Machine)

SVM are kernel-based ML models that define decision boundaries. As the number of properties increases, the decision boundary becomes higher order, called hyperplane [37].

The fundamental reason for using SVM is to separate numerous classes in the training data using a surface that maximizes the margin between them. In other words, SVM allows a model's generalization ability to be maximized. This is the goal of the Structural Risk Minimization principle (SRM), which allows for the minimization of a bound on a model's generalization error rather than minimizing the mean squared error on the set of training data, which is the commonly used by empirical risk minimization methods [38].

Definition S4.
Step 1: The hyperplane is defined.

$$y_i = \omega^T x_i + b \tag{14}$$

where, ω is a vector, and b is an offset between the origin plane and the hyperplane.

Definition S5.
Step 2: Transform the objective function into a double optimization.

$$min \frac{1}{2} \|\omega\|^2 \tag{15}$$

$$y_i(\omega^T x_i + b) \geq 1, i = 1, 2, \dots , n \tag{16}$$

where (x_i, y_i) is the sample data, x_i is the input variable, and y_i is the output variable, and the target hyperplane is found by solving the ω^T and b values. SVM for regression is essentially the same as SVM for classification. It is to discover the hyperplane with the maximum data. Insensitive loss parameters, as stated in Formula (17), are introduced to aid in hyperplane selection. where is the parameter for insensitivity loss.

Definition S6.

$$|y_i - \omega^T x_i - b| \leq \in, i = 1, 2, \dots, n \tag{17}$$

In actual applications, the model is frequently enhanced to deal with noise data by including penalty parameter C and slack variables $\xi_i^1,\ \xi_i^2$ [39].

Definition S7.

$$\min \frac{1}{2}(\omega)^2 + C \sum_{i=1}^{N} \xi_i^1 + \xi_i^2 \tag{18}$$

$$-\int -\xi_i^1 \leq y_i - \omega^T x_i - \mathrm{b} \leq \int +\xi_i^2, \xi_i^1 \geq 0, \xi_i^2 \geq 0 i = 1, 2, \dots, \mathrm{n} \tag{19}$$

2.4. LR (Logistic Regression)

LR is a mathematical model that estimates the likelihood of belonging to a specific class. The LR model is used for binary classification in this paper, but it can easily be extended for multi label classification in other cases. The formula for linear estimation is expressed as follows [40].

Definition S8.

$$g(z) = \frac{1}{(1+e^{-z})} \tag{20}$$

Definition S9.

The following is the definition of the linear boundary:

$$z = \theta^T x = \theta_0 x_0 + \theta_1 x_1 + \cdots + \theta_n x_n = \sum_{i=0}^{n} \theta_i x_i \ = \tag{21}$$

Definition S10.

The training data vector is $\mathrm{x} = [\mathrm{x}_0, \mathrm{x}_1, \mathrm{x}_2, \mathrm{x}_3, \dots, \mathrm{x}_n]^\tau$ and the optimum parameter is $\theta = [\mathrm{x}_0, \mathrm{x}_1, \mathrm{x}_2, \mathrm{x}_3, \dots, \mathrm{x}_n]^\tau$. The following is the prediction function:

$$h_\theta(x) = g(\theta^T x) = \frac{1}{1 + e^{-\theta^T x}} \tag{22}$$

Definition S11.

The above function's value represents the likelihood of $y = 1$. As a result, the odds that x belongs to class *1* and class *0* are stated as follows:

$$P(y = 1\ x; \theta) = h_0(x) \tag{23}$$

$$P(y = 0\ x; \theta) = 1 - h_0(x) \tag{24}$$

2.5. LSTM (Long Short-Term Memory)

Recurrent neural networks (RNN) with LSTM have emerged as an effective and scalable solution for various learning problems using sequential data [41]. Because they are broad and practical and excellent for capturing long-term temporal dependencies. The LSTM is an RNN-style architecture with gates that regulate information flow between cells. The input and forget gate structures can modify the information as it travels along the cell state, with the eventual output being a filtered version of the cell state based on the input context [42]. The mathematical expression for the LSTM algorithm is [43]:

Definition S12.

The input gate is expressed as

$$i_t = \sigma(w_i * [h_{t-1}, x_t] + b_i) \tag{25}$$

It determines whatever information from the previous cell can be passed to the current cell. The forget gate is described by equation (26), and it is used to save information from prior memory input or otherwise.

Definition S13.

$$f_t = \sigma(w_f * [h_{t-1}, x_t] + b_f) \tag{26}$$

Definition S14.

The cell's update is controlled by the control gate, which is specified as:

$$C_t = \tanh(w_c * [h_{t-1}, x_t] + b_c \tag{27}$$

$$C_t = f_t * C_{t-1} + i_t * C_t \tag{28}$$

Definition S15.

Finally, the output gate is used to update the hidden layer (h_{t-1}) and the output, which is determined by the following equations:

$$o_t = \sigma(w_c * [h_{t-1}, x_t] + b_0 \tag{29}$$

$$h_t = o_t * \tanh(C_t) \tag{30}$$

In the following equation, x_t represents input, w represents the associated weight matrix of input, b represents the corresponding bias of input, C_{t-1} represents previous block memory, C_t represents current block memory, h_{t-1} represents previous block output, and h_t represents current block output. Furthermore, tanh is the hyperbolic tangent function, which is utilized to scale values ranging from -1 to 1, and σ is the sigmoid activation function, which produces values ranging from 0 to 1. The LSTM algorithms were implemented as follows [43].
